# Factors Modulating the Occurrence of the Selective-Value Effect in Tufted Capuchin Monkeys (*Sapajus* spp.)

**DOI:** 10.3390/ani15030453

**Published:** 2025-02-06

**Authors:** Alessandra D’Onofrio, Serena Gastaldi, Elsa Addessi

**Affiliations:** 1 Dipartimento di Biologia Ambientale, Sapienza Università di Roma, 00185 Rome, Italy; alessandra.donofrio@outlook.com; 2 Istituto di Scienze e Tecnologie della Cognizione, Consiglio Nazionale delle Ricerche, 00197 Rome, Italy; serena.gastaldi@istc.cnr.it

**Keywords:** foraging theory, selective-value effect, less-is-better effect, capuchin monkeys, rational choice, food preference

## Abstract

During foraging decisions, animals often make irrational choices. The selective-value effect refers to the lack of preference for an option consisting of one highly preferred item plus one less preferred item compared to a single highly preferred item. A similar bias is the less-is-better effect, in which individuals prefer a single highly preferred item rather than an option that includes both a highly preferred item and a less preferred one. Here, we investigated the occurrence of these decisional biases in twelve tufted capuchin monkeys (*Sapajus* spp.) by exploring, for the first time in this genus, both the role of the relative value of the highly vs. less preferred food and the length of the intertrial interval. Overall, capuchins made rational choices by preferring the mixed option over the single one. However, on an individual level, some capuchins exhibited the selective-value effect. Mirroring previous findings in apes, both the relative value of the highly vs. less preferred food and the length of the intertrial interval modulated capuchins’ preference for the mixed option. This study provides further insight into the factors influencing violations of rationality in primate food choices, suggesting that these decisions might reflect cognitive adaptations for optimizing sensory feedback and nutrient intake rather than being irrational.

## Simple Summary

Just like human beings, animals often make irrational choices; that is, when making a choice, they do not maximize the amount of food they can obtain. The selective-value effect occurs when individuals show no distinct preference for high-value food when it is coupled with low-value food compared to when it is presented alone. In the present study, we investigated the occurrence of the selective-value effect in twelve tufted capuchin monkeys (*Sapajus* spp.) by exploring, for the first time in this genus, both the role of food quality and the time available to consume the food. Overall, capuchins preferred the more abundant food option over the single option. However, at the individual level, some capuchins showed the selective-value effect; that is, they did not show a preference for either option. Both food quality and the time available to consume the food modulated capuchins’ preferences. Our findings suggest that apparent irrational decisions might stem from sensory feedback and the need for nutrient intake maximization.

## 1. Introduction

Optimal foraging theory predicts that animals adopt behaviors that maximize their efficiency in finding and selecting food [1,2,3]. In this framework, it is expected that animals will always choose according to the tenets of economic rationality [4], i.e., to maximize their gains by prioritizing resources that optimize energy intake, both qualitatively and quantitatively [5,6,7]. However, in some contexts, animals may respond to external stimuli with behaviors that violate economic rationality [8]. For instance, even though animals are expected to prefer larger quantities of food over smaller ones, this is not systematically observed [8]. In this respect, two decisional biases have been described in both the human and non-human animal literature: the selective-value effect and the less-is-better effect [9,10,11,12,13,14,15,16].

When choosing between a more abundant option containing both a highly preferred item and a less preferred one, compared with an option consisting solely of the highly preferred item, subjects could show a lack of preference for either option [11,14]. According to Silberberg et al. [11], this occurs because individuals assign a value only to the highly preferred item within a mixed option, and this bias has been labeled as the “selective-value effect”. However, in the same choice context, individuals may rate the single highly preferred item as better than the larger alternative that includes both a highly preferred item and a less preferred one. This suboptimal choice behavior has been labeled as the “less-is-better effect” [10,13,15,16]. According to Kralik et al. [13], this occurs because the preference for an option depends on the overall value of its items; the addition of a less preferred item reduces the overall value of the combination. Thus, in both these decisional biases, an individual’s choice is influenced more by the perceived overall value of the items than by their quantity, and the presence of a less desirable item can diminish the appeal of the more abundant option.

Several studies have explored the occurrence of both the selective-value effect and the less-is-better effect in non-human animal species, including great apes (chimpanzees, *Pan troglodytes*; bonobos, *Pan paniscus*; Western gorillas, *Gorilla gorilla*; Sumatran orangutans, *Pongo abelii* [11,12,14]), Japanese macaques (*Macaca fuscata*; [11]), Rhesus macaques (*Macaca rhesus*; [13]), tufted capuchin monkeys (*Sapajus* spp.; [17,18]), dogs (*Canis lupus familiaris*; [19,20]), and pigeons (*Columba livia domestica*; [21]). Chimpanzees, bonobos, Western gorillas, Sumatran orangutans, Japanese macaques, and tufted capuchin monkeys showed the selective-value effect [11,12,14,17,18]. In some conditions, they did not show a preference for a mixed option consisting of two food units (a highly preferred food item plus a less preferred food item) over a single highly preferred food item. The occurrence of the selective-value effect was modulated by the relative value of highly and less preferred foods, and intertrial interval (ITI, defined as the time elapsed between two consecutive trials). When preferences between foods were very different, subjects preferred the mixed option to a lower extent than when preferences were more similar [14]. A similar trend was observed when ITIs were short, suggesting that the presence of less preferred food in the mixed option assumed a negative value since it was associated with a delay in completing the trial and thus having another opportunity to consume the highly preferred food [12]. However, the effect of ITIs was not consistent across studies: whereas Beran et al. observed this effect, both Sanchez-Amaro et al. [14] and Quintiero et al. [18] did not.

Rhesus macaques, capuchins, pigeons, and dogs [13,18,19,20,21] showed the less-is-better effect. Specifically, they preferred a single highly preferred food item over a mixed option containing both the highly preferred food and a less preferred food item, even when controlling for potentially confounding factors such as aversion to multiple options or food neophobia [20]. In Rhesus macaques, this effect was observed both in captivity and in the wild [13]. In pigeons, it was modulated by the level of food motivation and was observed only when birds were not food-restricted, resulting in a relatively low food motivation level [21].

The present study aims to investigate whether either the selective-value effect or the less-is-better effect occurs in tufted capuchin monkeys and which factors modulate the occurrence of these decisional biases. Capuchin monkeys are particularly well positioned for this research because they have previously shown several other decisional biases (e.g., framing effect [22]; endowment effect [23]; decoy effect [24,25,26]). The two previous studies that have investigated the occurrence of these decisional biases in capuchin monkeys were limited to just a few individuals: Huntsberry et al. [17] observed the selective-value effect in three capuchins, and Quintiero et al. [18] described the selective-value effect in two subjects and the less-is-better effect in one subject from their sample of eight capuchins. Additionally, they were unable to observe an effect related to intertrial interval length nor did they evaluate the role of the qualitative difference between food pairs.

Specifically, we explored how differences in preference levels between two types of food (small or large) affect decision-making processes, as well as the role of intertrial interval length (short or long) while controlling for potential confounding factors such as aversion to heterogeneity, quantitative discrimination failure, and food preferences. In line with the existing literature, we expected capuchins to choose the mixed option more frequently when qualitative differences between the two food items were small, indicating rational behavior. Conversely, when qualitative differences were large, we anticipated that they would either show indifference between options (selective-value effect) or prefer the single-item option (less-is-better effect) [14]. Additionally, we expected capuchins to choose the mixed option more often during longer ITIs rather than shorter ones [12], allowing them to consume selected foods more slowly. The results of this investigation can shed further insight into the range of decisional biases that share common evolutionary roots across the primate order, as well as on the factors that promote or limit their occurrence.

## 2. Materials and Methods

### 2.1. Subjects

We tested twelve captive tufted capuchin monkeys, consisting of six males and six females (average age: 24 years, minimum age 12 years; maximum age: 36 years), belonging to four different social groups (see Table 1), housed at the Primate Center of the Istituto di Scienze e Tecnologie della Cognizione (ISTC-CNR) in Rome.

The capuchins were housed in enriched indoor (25.4 m^3^) and outdoor areas (53.2 m^3^–127.4 m^3^, depending on the group size) and individually tested in experimental boxes measuring 1 m^3^ with free access to one of the two indoor areas, with water available ad libitum. Animals were not deprived of food for testing.

Experimental sessions were conducted between 9:15 a.m. and 2:30 p.m., prior to their daily meal. Their meal occurs at 3:00 p.m. and consists of a wide variety of fruits, vegetables, and monkey chow. We tested individual subjects on a voluntary basis, and we separated them from the rest of the group by offering small amounts of food (peanut seeds) as reinforcement. The study was carried out in accordance with the European normative regulating the use of primates in experimental research (Directive 2010/63/EU; authorization n. 721/2024-PR to Elsa Addessi) and the guidelines of the OPBA (Animal Welfare Board) operating at the ISTC-CNR Primate Center.

### 2.2. Experimental Apparatus and Procedure

**Figure 1:**
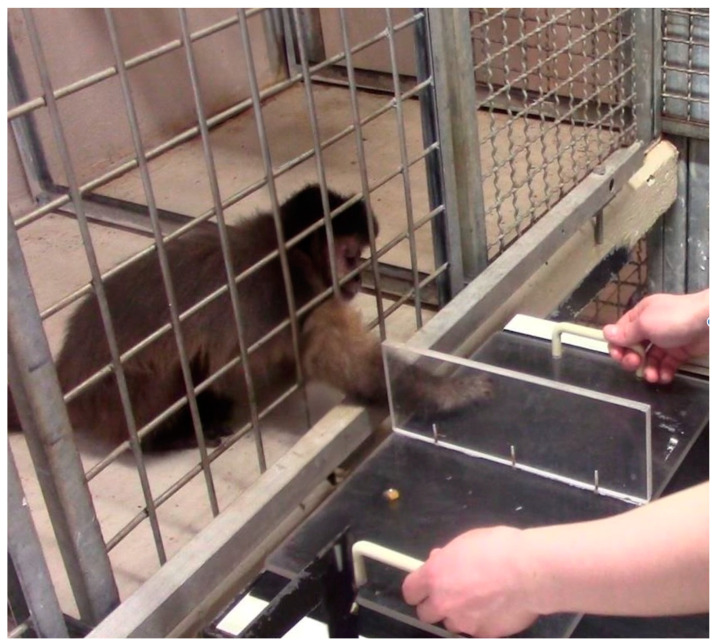
This picture depicts a female capuchin choosing the mixed option (not visible) during an experimental trial of the “HL+30” condition.

In each experimental phase, animals were offered binary choices on a transparent Plexiglas tray (27 × 40 cm), featuring a central divider of the same material and two handles on each side. Food items were placed 5.2 cm from the top edge of the apparatus and 8 cm from the tray divider (Figure 1).

The choice options were positioned on the tray out of the subject’s sight. The tray was placed on a black trolley situated between the experimenter and the subject’s experimental box. In each trial, the tray was slid towards the subject when it was positioned in front of the trolley. The tray remained in place for up to 15 seconds until the subject chose one of the two alternatives. The experimenter avoided eye contact with the subject to prevent biasing its choice and did not look at either of the two options. Each subject made its choice by inserting a hand through the wire mesh to pick up one food option. Once a choice was made, the experimenter immediately removed the tray to prevent the subject from picking up the alternative food option. Choices made by each subject were recorded during the experimental sessions, which were also videotaped. In the food preference phase (described in Section 2.3), there was a 10 s intertrial interval (ITI). During the experimental phase (described in Section 2.4), there was either a 10 s or a 30 s ITI, depending on the condition.

### 2.3. Food Preference Phase

First, we selected two pairs of differently preferred food items: (i) a highly preferred (H) vs. an intermediate-preferred (L+) pair, in which H was selected over L+ in 65–75% of the trials across two consecutive 20-trial sessions; (ii) a highly preferred (H) vs. a low-preferred (L−) pair, in which H was selected over L− in 95–100% of the trials across two consecutive sessions. Each subject performed one twenty-trial session per day.

In each trial of the food preference phase, subjects were provided with the chosen food item. Immediately after each session involving the highly preferred (H) vs. low-preferred (L−) food pair, we conducted a palatability test to assess whether subjects would consume the low-preferred food (L−), despite not preferring it. For this test, we offered L− food for 10 trials (one item per trial) and assessed whether capuchins ate each food item within 15 seconds. All capuchins consumed the L− food in all trials except for two individuals: one ate the L− food in 8 out of 10 trials, while another did so in 9 out of 10 trials.

The food types that could be cut into small pieces (e.g., pineapple, apricot, monkey chow, raisin, plum) were all uniform in size, while other foods (e.g., Rice Krispies, sunflower seeds, Cheerios, pumpkin seeds) were presented in their entirety. The weight of each item was carefully standardized using a precision scale (Gibertini EUROPE 1700, dd = 0.01 g, SER. NO82998). The weights were as follows: monkey chow—0.02 g; Rice Krispies—0.01 g; sunflower seeds—0.03 g; Cheerios—0.10 g; apricot—0.30 g; raisin—0.20 g; pineapple—0.30 g; plum—0.30 g; pumpkin seeds—0.05 g. Table S1 summarizes all individual food preferences.

### 2.4. Experimental Phase

#### 2.4.1. Experimental Conditions

To test whether the relative value of the highly vs. less preferred food in each pair (highly preferred H vs. intermediate-preferred L+ and highly preferred H vs. low-preferred L−) and/or the length of the ITI (10 seconds or 30 seconds) modulates the occurrence of the selective-value effect, subjects were tested under four conditions: (i) “HL+10”, in which the highly preferred food (H) and the intermediate-preferred food (L+) were presented with a 10-second ITI; (ii) “HL−10”, in which the highly preferred food (H) and the low-preferred food (L−) were presented with a 10-second ITI; (iii) “HL+30”, in which the highly preferred food (H) and the intermediate-preferred food (L+) were presented with a 30-second ITI; (iv) “HL−30”, in which the highly preferred food (H) and the low-preferred food (L−) were presented with a 30-second ITI.

Subjects were first tested under conditions “HL+10” and “HL−10”, with their order of presentation counterbalanced across subjects, followed by conditions “HL+30” and “HL−30”, again counterbalancing their order across subjects. In each condition, subjects completed five sessions. The entire experiment was then replicated a second time (see Table S2). Thus, each subject participated in two blocks of four five-session conditions, resulting in a total of forty sessions, with one session conducted each day.

#### 2.4.2. Experimental Sessions

Each experimental session consisted of twenty-four trials, which included (1) six experimental trials (HL+ vs. H and HL− vs. H), in which a single unit of highly preferred food (H) was presented against a mixed option consisting of H and the less preferred food (L+ in conditions “HL+10” and “HL+30”; L− in conditions “HL−10” and “HL−30”); (2) six control trials for heterogeneity aversion (HL+ vs. L+ and HL− vs. L−), in which a single unit of less preferred food (L+ in conditions “HL+10” and “HL+30”; L− in conditions “HL−10” and “HL−30”) was presented against a mixed option consisting of highly preferred food (H) and less preferred food (L+ in conditions “HL+10” and “HL+30”; L− in conditions “HL−10” and “HL−30”); (3) three control trials for quantitative discrimination with highly preferred food H, in which a single unit of H was presented against two units of H (HH vs. H), and three control trials for quantitative discrimination with less preferred food L (L+L+ vs. L+ and L−L− vs. L−), in which a single unit of L+ or L−, depending on the condition (see above), was presented against two units of either L+ or L−, and (4) six control trials for food preference maintenance (H vs. L+ and H vs. L−), in which a single unit of highly preferred food (H) was presented against a single unit of less preferred food (L+ in conditions “HL+10” and “HL+30”; L− in conditions “HL−10” and “HL−30”). Each subject received a total of nine hundred and sixty trials across the two blocks. Due to experimenter error, in four sessions of the “HL−10” condition all subjects received seven heterogeneity aversion control trials (HL− vs. L−) and five food preference maintenance control trials (H vs. L−), and in one session of the “HL+10” condition three subjects received seven food preference maintenance control trials (H- vs. L+) and five experimental trials (HL+ vs. H).

In heterogeneity aversion trials, we assessed whether capuchins showed an aversion to choosing the mixed option—composed of two different food items—regardless of their preferences. In quantitative discrimination trials, we evaluated whether capuchins could correctly discriminate between different quantities of food items. In food preference maintenance trials, we examined whether capuchins maintained their preferences over time.

In each trial type, the positions of the two food options on the tray (right/left) were pseudo-randomized within each experimental session. Before each experimental session began, subjects were offered one item each from both food types to consume.

### 2.5. Data Analysis

To determine whether capuchins significantly preferred either option above chance level, for each trial type (experimental, heterogeneity aversion, quantitative discrimination, and food preference maintenance), we employed the single-sample Wilcoxon signed-ranks test at both the population and individual levels.

Next, to evaluate the two predictors of interest—the relative value of the highly vs. less preferred food and the length of the intertrial interval—we implemented random-effects logistic regression models for each trial type. Regression methods for longitudinal data analysis are particularly suited for analyzing behavioral and ecological data. These models account for interdependency and structuring of the data and thus allow the use of multiple data points from the same subject while avoiding the problem of pseudoreplication [27,28].

As dependent variables, we used choices for the mixed option (HL+ and HL−) in experimental and heterogeneity control trials; choices for the more numerous option (HH, L+L+ or L−L−) in quantitative discrimination control trials; and choices for the highly preferred food option (H) in food preference maintenance control trials. The type of less preferred food (L+ or L−) and ITI (10 seconds or 30 seconds) were included as factors, while sex, age, and block (i.e., first or second part of the experiment, see Section 2.4.1 and Table S2) were included as covariates. For all regressions, the identity of each subject was included as a random effect. Interactions were analyzed using the Wald test; non-significant interactions were removed from the model before repeating the analysis. The significance level was set at *p* < 0.05. We conducted this analysis using Stata 14.0 software.

## 3. Results

### 3.1. Experimental Trials (HL+ vs. H; HL− vs. H)

The capuchins significantly preferred the mixed option, consisting of the highly preferred food H plus the less preferred food (L+ in the conditions “HL+10” and “HL+30”, L− in the conditions “HL−10” and “HL−30”) above chance level in all experimental conditions (see Table 2). However, at the individual level, some subjects showed a lack of preference for either option, thus showing the selective-value effect. In the condition “HL+10”, one out of 12 subjects (Paprika) showed the selective-value effect; in “HL−10”, eight out of 12 subjects showed it (Cognac, Gal, Paprika, Robinia, Robiola, Robot, Saroma, Vispo); in “HL+30”, no subject showed the selective-value effect; however, in “HL−30”, five out of 12 subjects showed it (Robinia, Robiola, Robot, Saroma, Vispo) (Table S3).

**Figure 2:**
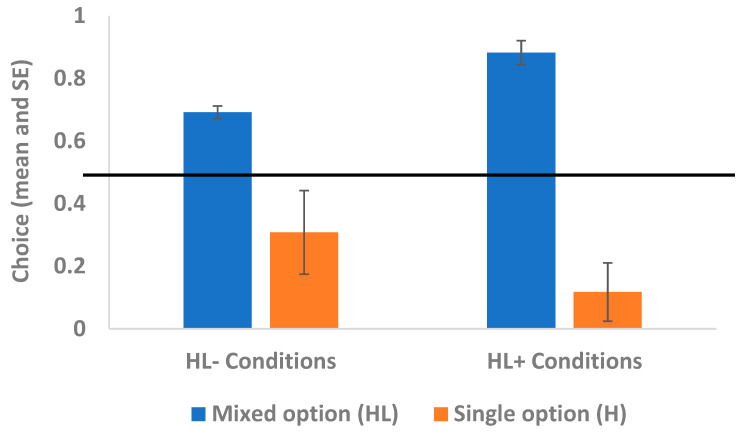
Experimental trials: effect of relative value of the highly vs. less preferred food. The figure depicts capuchins’ choices for the mixed option (consisting of the highly preferred food H plus the less preferred food L+ or L−, according to the condition; see Section 2.4.2). Capuchins significantly chose the mixed option more in the conditions involving the intermediate-preferred food L+ (“HL+10” and “HL+30”) than in the conditions involving the low-preferred food L− (“HL−10” and “HL−30”). The solid black line depicts the chance level.

**Figure 3:**
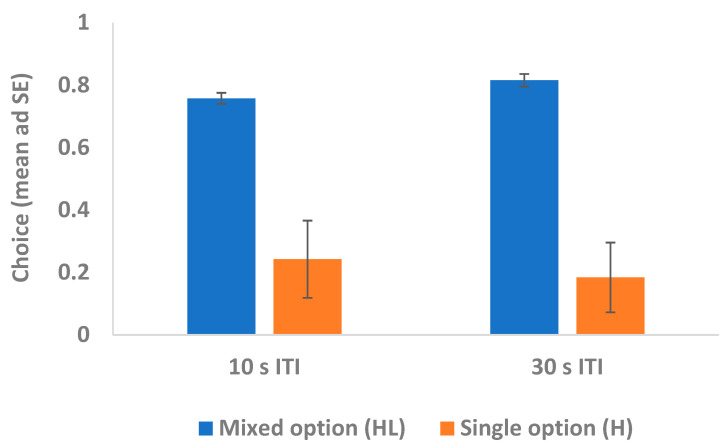
Experimental trials: effect of the ITI. The figure depicts capuchins’ choices for the mixed option (consisting of the highly preferred food H plus the less preferred food L+ or L−, according to the condition; see Section 2.4.2). Capuchins significantly chose the mixed option more in the conditions involving a longer ITI (“HL+30” and “HL−30”) than in the conditions involving a shorter ITI (“HL+10” and “HL−10”).

The random-effects logistic regression model showed no significant interactions (condition x ITI: χ^2^_2_ = 0.60, *p* = 0.438; condition x block: χ^2^_2_ = 0.31, *p* = 0.580), but there were significant main effects of condition and ITI. Capuchins showed a significantly greater preference for the mixed option when it included the intermediate-preferred food (L+) (i.e., in the conditions “HL+10” and “HL+30”) compared to when it included the low-preferred food (L−) (i.e., in the conditions “HL−10” and “HL−30”; see Figure 2) (coeff = −1.273; z = −3.41; *p* = 0.001). Moreover, capuchins showed a significantly greater preference for the mixed option when the ITI was longer (i.e., in the conditions “HL+30” and “HL−30”) than when the ITI was shorter (i.e., in the conditions “HL+10” and “HL−10”; see Figure 3) (coeff = 0.379; z = 2.50; *p* = 0.012). There were no other significant main effects.

### 3.2. Control Trials for Heterogeneity Aversion (HL+ vs. L+; HL− vs. L−)

When presented with heterogeneity aversion control trials (see Section 2.4.2), capuchins significantly preferred the mixed option above chance level (see Table 3). At the individual level, all subjects (except for Gal in the condition “HL+10”) preferred the mixed option over the single option (Table S4).

The random-effects logistic regression model showed no significant interactions (condition x ITI: χ^2^_2_ = 0.02, *p* = 0.882; condition x block: χ^2^_2_ = 3.24, *p* = 0.072), but it did reveal a significant main effect of condition. Capuchins chose the mixed option significantly more when it included the low-preferred food (L−) (i.e., in the conditions “HL−10” and “HL−30”) than when it included the intermediate-preferred food (L+) (i.e., in the conditions “HL+10” and “HL+30”) (coeff = 2.296, z = 4.63, *p*< 0.001). There were no other significant main effects.

### 3.3. Control Trials for Quantitative Discrimination (HH vs. H; L+L+ vs. L+; L− L− vs. L−)

When presented with quantitative discrimination control trials (see Section 2.4.2), capuchins overall significantly preferred the larger option, which consisted of two highly preferred (HH), intermediate-preferred (L+L+), or low-preferred (L−L−) food items over the suboptimal alternative, which consisted of a single unit of highly preferred (H), intermediate-preferred (L+), or low-preferred (L−) food in all quantitative discrimination trial types (see Table 4). Individual results confirmed these findings (Tables S5 and S6).  

For the trials involving the highly preferred food (H), the random-effects logistic regression model showed no significant interactions (condition x ITI: χ^2^_2_ = 0.05, *p* = 0.826; condition x block: χ^2^_2_ = 0.28, *p* = 0.599), but there was a significant main effect of condition. Capuchins preferred the larger option (two units of highly preferred food) significantly more in the conditions “HL−10” and “HL−30” (coeff = 1.086; z = 3.43; *p* = 0.001). For the trials involving the less preferred food, there was a significant interaction between food type (L+ or L−) and ITI (10 seconds or 30 seconds): χ^2^_1_ = 10.93, *p* < 0.001. Post hoc analysis showed that capuchins preferred the larger option (two units of less preferred food) significantly more in the condition “HL+30” (i.e., with the intermediate-preferred food L+) than in the condition “HL−30” (i.e., with the low-preferred food L−) (coeff = 1.201; z = 2.64; *p* = 0.008). However, there was no significant difference between the conditions “HL+10” and “HL−10” (coeff = −0.065; z = −0.12; *p* = 0.907). There were no other significant interactions or main effects.

### 3.4. Control Trials for Food Preference Maintenance (H vs. L+; H vs. L−)

When presented with food preference maintenance control trials (see Section 2.4.2), in the conditions “HL+10” and “HL+30”, capuchins did not significantly prefer the highly preferred food (H) above chance level, whereas they did so in the conditions “HL−10” and “HL−30” (see Table 5). At the individual level, many subjects showed a lack of preference for either option or had reversed their preferences. In the condition “HL+10”, three out of twelve subjects were indifferent between options (Robiola, Sandokan, Saroma) and four subjects had reversed their food preferences (Gal, Roberta, Rucola, Vispo). Similarly, in the condition “HL+30”, seven out of twelve subjects were indifferent between options (Gal, Paprica, Robinia, Robiola, Rucola, Saroma, Totò), and two subjects had reversed their preferences (Roberta and Vispo). Finally, in the condition “HL−30”, one subject (Totò) was indifferent between options (Table S7).

The random-effects logistic regression model showed a three-way significant interaction between food type (L+ or L−), ITI (10 seconds or 30 seconds), and block (first part of the experiment vs. second part of the experiment, see Section 2.4.1 and Table S2): χ^2^_2_ = 15.16, *p* < 0.001. Post hoc analyses revealed that, in H vs. L+ trials, capuchins chose the H food over the L+ food significantly more in the first part than in the second part of the experiment (coeff = 0.410; z = 3.30; *p* < 0.001), regardless of ITI (coeff = 0.103; z = 0.84; *p* = 0.404). In contrast, in H vs. L− trials, capuchins chose the H food over the L− food significantly more in the second part than in the first part of the experiment (coeff = 0.740; z = 3.01; *p* = 0.003), and significantly more in trials with a 10-second ITI than with a 30-second ITI (coeff = 0.471; z = 1.97; *p* = 0.049).

## 4. Discussion

Overall, capuchins made rational choices, as they significantly preferred the mixed option above chance level during the experimental trials. However, both the relative value of the highly preferred vs. less preferred food included in the mixed option and the length of the intertrial interval (ITI) independently influenced their choices. Regardless of the ITI, the preference for the mixed option was stronger when the highly and less preferred food items were similarly favored (i.e., in the “HL+” conditions) than when the two food items were rated as more qualitatively different by capuchins (i.e., in the “HL−” conditions), as shown by Sánchez-Amaro et al. [14] in great apes. Moreover, regardless of the relative difference between food items, the preference for the mixed option was stronger when the ITI was longer compared to when it was shorter (i.e., in conditions with 30-second ITI), as reported by Beran et al. [12] in chimpanzees.

From the individual performances, it emerged that many capuchins exhibited the selective-value effect; that is, they were indifferent between the mixed option and the single option in conditions involving two very differently preferred foods. This indifference was more pronounced when the ITI was 10 seconds compared to when it was 30 seconds. Specifically, out of twelve capuchins, eight displayed the selective-value effect in the “HL−10” condition, while five did so in the “HL−30” condition. In contrast, only one capuchin showed the selective-value effect in the “HL+10” condition, and none did so in the “HL+30” condition. This rate is higher than that reported by Quintiero et al. [18], in which only two out of eight capuchins exhibited the selective-value effect, and one demonstrated the less-is-better effect. This discrepancy may be attributed to the overall lower number of trials in that study (only 40 trials compared to the 240 experimental trials conducted in the present investigation).

As Sánchez-Amaro et al. [14] suggested, when the difference in the relative value between foods is low, individuals may perceive the choice between the mixed and single options as quantitative discrimination, focusing on the larger amount of food while disregarding the qualitative differences between the foods. However, when the difference in the relative value between the foods included in the mixed option is more substantial, the less preferred food might lose its value [11], leading individuals to choose randomly between options.

This interpretation aligns with the results of the preference maintenance control trials (H vs. L+ and H vs. L−), which confirmed that capuchins’ relative preference for the food pairs H vs. L+ (highly preferred vs. intermediate preferred) and H vs. L− (highly preferred vs. low-preferred) was different. Overall, regardless of the ITI, in the conditions “HL+10” and “HL+30”, in which the difference in the relative value between foods was small (i.e., the H food was preferred over the L+ food in 65–75% of the trials during the preliminary food preference phase), capuchins did not significantly choose the highly preferred food above chance level, and their preference for the intermediate-preferred L+ food increased over time. In contrast, in the conditions “HL−10” and “HL−30”, in which the difference in the relative value between foods was higher (i.e., the H food was preferred over the L− food in 95–100% of the trials during the preliminary food preference phase), capuchins significantly chose the highly preferred food above chance level, and their preference for the low-preferred L− food decreased over time. Accordingly, at the individual level, in the conditions “HL+”, many subjects showed a lack of preference for either option or had reversed their preferences: out of twelve capuchins, in the condition “HL+10”, three were indifferent between options and four had reversed their preferences; in the condition “HL+30”, seven capuchins were indifferent between options and two had reversed their preferences. In contrast, in the condition “HL−10”, all capuchins continued to prefer the H food, while in the condition “HL−30”, only one subject was indifferent between options.

The modulation of capuchins’ choices for the mixed option by the length of the intertrial interval—specifically that capuchins preferentially selected the mixed option when the ITI was 30 seconds rather than 10 seconds—suggests that capuchins prefer to choose the mixed food option (HL) over a single unit of the highly preferred food (H) when they have more time available to consume the more abundant option before the subsequent trial, thus allowing another opportunity to consume the highly preferred food. In contrast, they chose the mixed option less frequently when the intertrial interval was shorter, likely to avoid excessively delaying the occurrence of the next trial [12]. This hypothesis is supported by observations that wild environmental pressures, such as predation or competition, may reduce the time available for foraging and drive animals to prioritize specific, high-value resources [13,29].

However, a qualitative comparison of the regression coefficients shows that the relative value of the highly preferred food compared to the less preferred food was a slightly stronger modulator of capuchins’ choices for the mixed over the single option than the length of the intertrial interval. Additionally, the analysis of each capuchin’s choices, showing that eight capuchins exhibited the selective value-effect in the condition “HL−10”, while only five did so in the condition “HL−30” (as reported above), corroborates this finding. This aligns with previous results showing that the effect of the length of the intertrial interval on the occurrence of the selective-value effect is labile; it was observed in some studies i.e., [12], but not in others i.e., [14,18]. In an ecological context, an animal may not only strive for efficiency in energy intake relative to time but may also forage based on specific nutrient needs [5]. Individuals may vary their provisioning strategies in relation to their metabolic homeostasis. For instance, Izar et al. [30] observed a fixed and constant daily protein intake pattern (protein prioritization) in natural populations of bearded capuchins (*Sapajus libidinosus*), a phenomenon also demonstrated in other primate species, including humans [31,32,33]. According to this model, variations in protein intake can influence the consumption of other macronutrients, such as fats and carbohydrates, whose quantities may vary daily. It is possible that capuchins prioritize certain foods while avoiding others without these decisions being suboptimal. This prioritization may depend not only on physiological feedback generated by food resources [29,33], but also on individual preferences and sensory cues, that precede physiological feedback [34].

We can exclude the possibility that the selective-value effect observed in several capuchins was due to heterogeneity aversion or difficulties with quantitative discrimination. In control trials for heterogeneity aversion (HL+ vs. L+ and HL− vs. L−), capuchins significantly preferred the mixed option over the single, less preferred food. In control trials for quantitative discrimination (HH vs. H, LL+ vs. L+, LL− vs. L−), capuchins chose the larger option significantly above chance. However, in heterogeneity trials under “HL+” conditions, capuchins selected the mixed HL option less often than in “HL−” conditions, likely because their choices were influenced by the single unit of the intermediate-preferred L+ food, which was qualitatively similar to the highly preferred food. Similarly, in “HL+” conditions, capuchins chose the two units of highly preferred food (HH) less frequently than in “HL−” conditions, probably to balance their higher selection of the less preferred food option, especially in the “HL+30” condition (L+L+) compared to the “HL−30” condition (L−L−).

Overall, our findings indicate that in experimental trials, capuchin monkeys made rational choices, generally preferring the option yielding a larger food amount (i.e., the mixed option) over the single highly preferred food unit. However, as expected, the likelihood of choosing the mixed option was significantly lower when the two foods were very differently preferred and when the intertrial interval was shorter. In these conditions, many capuchins did not make rational choices and preferred the single highly preferred food unit over the mixed option that included both the highly preferred food and the low-preferred food. This behavior demonstrates the selective-value effect, similar to that observed in other non-human animals.

## 5. Conclusions

In conclusion, despite large interindividual differences, this study sheds further light on the factors modulating the occurrence of the selective-value effect and extends the results previously obtained in capuchin monkeys [17,18], expanding the range of decisional biases that share common evolutionary roots across the primate order. Understanding decisional biases in light of economic axioms can be misleading, and irrational behaviors—although not necessarily applicable to an entire population—should be analyzed through a biological lens. Indeed, natural selection does not operate under principles of coherence, unlike the behavioral models proposed by neoclassical economic theory. While some animal choices may appear irrational, they can be biologically rational as adaptive responses aimed at optimizing individual fitness [35,36,37].

The present study contributed to showing the external factors that potentially affect decisional biases and thus decrease choice rationality. Future studies should further investigate which individual characteristics may be associated with the occurrence of decisional biases, whether these biases also operate in wild populations, and to what extent they affect their fitness.

## Figures and Tables

**Table 1:** Subjects’ sex, age, species, and social group.

Subjects	Sex	Age	Species	Social Group
Gal	M	32	*S. nigritus*	1
Paprica	F	33	*S. paraguay/cay*	1
Totò	M	12	*S. paraguay/cay*	1
Robinia	F	29	*S. paraguay/cay*	2
Robot	M	27	*S. paraguay/cay*	2
Saroma	F	21	*S. paraguay/cay*	2
Roberta	F	36	*S. paraguay/cay*	3
Sandokan	M	22	*S. paraguay/cay*	3
Vispo	M	22	*S. macrocephalus*	3
Cognac	M	35	*S. apella*	4
Robiola	F	24	*S. paraguay/cay*	4
Rucola	F	22	*S. paraguay/cay*	4

**Table 2:** Experimental trials. The table reports the results of Wilcoxon’s single-sample test, including the *p* value, median, and interquartile range. Capuchins preferred the mixed option (consisting of the highly preferred food H plus the less preferred food L+ or L−, according to the condition, as detailed in Section 2.4.2) above chance level in all conditions.

Experimental Conditions	Trial Type	z	*p* Value	Median	Interquartile Range
“HL+10”	HL+ vs. H 10 s ITI	3.062	0.002	0.899	0.187
“HL−10”	HL− vs. H 10 s ITI	2.871	0.004	0.617	0.112
“HL+30”	HL+ vs. H 30 s ITI	3.061	0.002	0.917	0.125
“HL−30”	HL− vs. H 30 s ITI	2.982	0.003	0.725	0.292

**Table 3:** Control trials for heterogeneity aversion. The table reports the results of Wilcoxon’s single-sample test, including the *p* value, median, and interquartile range. Capuchins preferred the mixed option (consisting of the highly preferred food H plus the less preferred food L+ or L−, depending on the condition; see Section 2.4.2) above chance level in all conditions.

Experimental Conditions	Trial Type	z	*p* Value	Median	Interquartile Range
“HL+10”	HL+ vs. L+10 s ITI	3.064	0.002	0.900	0.104
“HL−10”	HL− vs. L− 10 s ITI	3.133	0.002	1.000	0.179
“HL+30”	HL+ vs. L+30 s ITI	3.069	0.002	0.967	0.158
“HL−30”	HL− vs. L− 30 s ITI	3.165	0.002	1.000	0.017

**Table 4:** Control trials for quantitative discrimination. The table reports the results of Wilcoxon’s single-sample test, including the *p* value, median, and interquartile range. Capuchins preferred the larger option, which consisted of two units of highly preferred food or two units of less preferred food (L+ or L−, depending on the condition; see Section 2.4.2) above chance level in all conditions.

Experimental Conditions	Trial Type	z	*p* Value	Median	Interquartile Range
“HL+10”	HH vs. H 10 s ITI	3.104	0.002	0.967	0.058
	L+L+ vs. L+10 s ITI	3.103	0.002	0.983	0.092
“HL−10”	HH vs. H 10 s ITI	3.126	0.002	0.983	0.033
	L−L− vs. L−10 s ITI	3.086	0.0022	0.950	0.093
“HL+30”	HH vs. H 30 s ITI	3.097	0.002	0.967	0.067
	L+L+ vs. L+30 s ITI	3.130	0.002	1.000	0.058
“HL−30”	HH vs. H 30 s ITI	3.140	0.002	1.000	0.033
	L−L− vs. L−30 s ITI	3.072	0.002	0.950	0.092

**Table 5:** Control trials for food preference maintenance. The table reports the results of Wilcoxon’s single-sample test, including the *p* value, median, and interquartile range. Capuchins did not significantly prefer the highly preferred food above chance level in the conditions “HL+10” and “HL+30”, but they did so in the conditions “HL−10” and “HL−30”.

Experimental Conditions	Trial Type	z	*p* Value	Median	Interquartile Range
“HL+10”	H vs. L+10 s ITI	0.471	0.638	0.526	0.654
“HL−10”	H vs. L−10 s ITI	3.074	0.002	0.977	0.094
“HL+30”	H vs. L+30 s ITI	0.942	0.346	0.625	0.304
“HL−30”	H vs. L−30 s ITI	3.086	0.002	0.983	0.096

## Data Availability

Data is contained within the article or Supplementary Materials.

## Supplementary Materials

The following supporting information can be downloaded at: https://www.mdpi.com/article/10.3390/ani15030453/s1: Table S1. Food preference phase; Table S2. Order of presentation of experimental conditions across subjects; Table S3. Experimental trials (HL+/− vs. H); Table S4. Control trials for heterogeneity (HL+/− vs. L+/−); Table S5. Control trials for quantitative discrimination (HH vs. H); Table S6. Control trials for quantitative discrimination (LL+/− vs. L+/−); Table S7. Control trials for food preference maintenance (H vs. L+; H vs. L−).
