# Supplementary material for: Factors Modulating the Occurrence of the Selective-Value Effect in Tufted Capuchin Monkeys (Sapajus spp.)
Source: Animals (Basel). 2025 Feb 6;15(3):453. doi: 10.3390/ani15030453 (PMC11815744; doi:10.3390/ani15030453)
Supplement: Supplementary file 1 [file animals-15-00453-s001.zip › animals-3416927-supplementary.pdf]

Supplementary materials

Factors modulating the occurrence of the selective-value effect in tufted capuchin monkeys (*Sapajus* spp.)

Alessandra D'Onofrio, Serena Gastaldi, Elsa Addessi

Table S1. Food preference phase. Individual preferences for highly preferred (H) *vs*. intermediate-preferred food (L+) and highly preferred (H) *vs*. low-preferred (L-) food items

	H<br>Highly preferred food	L+<br>Intermediate-preferred food	H<br>Highly preferred food	L-<br>Low-preferred food
Cognac	Cheerios	Sunflower seed	Dried Pineapple	Raisin
Gal	Plum	Cheerios	Dried Pineapple	Rice Krispies
Paprika	Dried Pineapple	Sunflower seed	Dried Pineapple	Rice Krispies
Roberta	Dried Apricot	Cheerios	Dried Pineapple	Cheerios
Robinia	Raisin	Cheerios	Raisin	Rice Krispies
Robiola	Dried Pineapple	Cheerios	Raisin	Monkey chow
Robot	Dried Pineapple	Cheerios	Dried Pineapple	Monkey chow
Rucola	Dried Pineapple	Raisin	Cheerios	Sunflower seed
Sandokan	Dried Pineapple	Raisin	Dried Pineapple	Sunflower seed
Saroma	Plum	Dried Apricot	Dried Pineapple	Dried Apricot
Totò	Dried Pineapple	Pumpkin seed	Dried Pineapple	Sunflower seed
Vispo	Dried Apricot	Cheerios	Dried Pineapple	Rice Krispies

Table S2. Order of experimental conditions presentation across subjects in the two blocks of the Experimental phase

"A" Group<br>(3 Females + 3 Males)<br>Gal, Paprika, Robot, Robinia, Saroma, Totò	Block 1	"HL+ 10"<br>x 5 sessions	"HL- 10"<br>x 5 sessions	"HL+ 30"<br>x 5 sessions	"HL- 30"<br>x 5 sessions
	Block 2	"HL+ 10"<br>x 5 sessions	"HL- 10"<br>x 5 sessions	"HL+ 30"<br>x 5 sessions	"HL- 30"<br>x 5 sessions
"B" Group<br>(3 Females + 3 Males)<br>Cognac, Roberta, Robiola, Rucola, Sandokan, Vispo,	Block 1	"HL- 10"<br>x 5 sessions	"HL+ 10"<br>x 5 sessions	"HL- 30"<br>x 5 sessions	"HL+ 30"<br>x 5 sessions
	Block 2	"HL- 10"<br>x 5 sessions	"HL+ 10"<br>x 5 sessions	"HL- 30"<br>x 5 sessions	"HL+ 30"<br>x 5 sessions

Table S3. Experimental trials (HL+ *vs*. H and HL- *vs*. H). The table reports the individual results of Wilcoxon's single-sample test, including the p value, median, and interquartile range. Significant results are highlighted in bold

	"HL^+ 10"	"HL^- 10"	"HL^+ 30"	"HL^- 30"
**Cognac**	**z=2.842, p=0.004, interquartile range=0.208**	z=1.864, p=0.062, interquartile range=0.208	**z=2.859, p=0.004, interquartile range=0.167**	**z=2.407, p=0.016, interquartile range=0.375**
**Gal**	**z=2.972, p=0.003, interquartile range=0.083**	**z=2.587, p=0.010, interquartile range=0.208**	**z=2.913, p=0.004, interquartile range=0.167**	**z=2.755, p=0.006, interquartile range=0.167**
**Paprika**	z=1.225, p=0.221, interquartile range=0.375	z=1.222, p=0.221, interquartile range=0.375	**z=2.850, p=0.004, interquartile range=0.333**	**z=2.783, p=0.005, interquartile range=0.333**
**Roberta**	**z=2.970, p=0.003, interquartile range=0.042**	z=2.377, p=0.017, interquartile range=0.333	**z=3.051, p=0.002, interquartile range=0.000**	**z=2.811, p=0.005, interquartile range=0.167**
**Robinia**	**z=2.792, p=0.005, interquartile range=0.167**	z=0.262, p=0.794, interquartile range=0.333	**z=2.842, p=0.004, interquartile range=0.333**	z=1.633, p=0.102, interquartile range=0.375
**Robiola**	**z=3.051, p=0.002, interquartile range=0.000**	z=1.255, p=0.210, interquartile range=0.500	**z=2.972, p=0.003, interquartile range=0.042**	z=-0.207, p=0.836, interquartile range=0.333
**Robot**	**z=2.889, p=0.004, interquartile range=0.167**	z=-1.215, p=0.224, interquartile range=0.167	**z=2.913, p=0.004, interquartile range=0.167**	z=1.344, p=0.179, interquartile range=0.250

**Rucola**	**z=3.051, p=0.002, interquartile range=0.000**	z=2.850, p=0.004, interquartile range=0.333	**z=2.873, p=0.004, interquartile range=0.208**	**z=2.970, p=0.003, interquartile range=0.042**
**Sandokan**	**z=2.823, p=0.005, interquartile range=0.333**	z=2.377, p=0.017, interquartile range=0.333	**z=2.850, p=0.004, interquartile range=0.208**	**z=2.970, p=0.003, interquartile range=0.042**
**Saroma**	**z=2.705, p=0.007, interquartile range=0.275**	z=1.526, p=0.127, interquartile range=0.167	**z=3.000, p=0.003, interquartile range=0.000**	z=1.615, p=0.106, interquartile range=0.250
**Totò**	**z=2.705, p=0.007, interquartile range=0.250**	z=2.836, p=0.005, interquartile range=0.333	**z=2.792, p=0.005, interquartile range=0.167**	**z=2.828, p=0.005, interquartile range=0.167**
**Vispo**	**z=3.162, p=0.002, interquartile range=0.000**	z=1.341, p=0.180, interquartile range=0.375	**z=3.162, p=0.002, interquartile range=0.000**	z=1.545, p=0.122, interquartile range=0.375

Table S4. Control trials for heterogeneity aversion (HL+ *vs.* L+ and HL- *vs.* L-). The table reports the individual results of Wilcoxon's single-sample test, including the p value, median, and interquartile range. Significant results are highlighted in bold

	**"HL^+ 10"**	**"HL^- 10"**	**"HL^+ 30"**	**"HL^- 30"**
**Cognac**	**z=2.919, p=0.003, interquartile range=0.1667**	**z=2.970, p=0.003, interquartile range=0.0417**	**z=3.162, p=0.002, interquartile range=0.000**	**z=3.162, p=0.002, interquartile range=0.000**
**Gal**	z=1.846, p=0.065, interquartile range=0.542	**z=3.051, p=0.002, interquartile range=0.000**	**z=2.836, p=0.005, interquartile range=0.333**	**z=3.162, p=0.002, interquartile range=0.000**
**Paprika**	**z=2.828, p=0.005, interquartile range=0.1667**	**z=2.913, p=0.004, interquartile range=0.1667**	**z=2.796, p=0.005, interquartile range=0.3333**	**z=3.162, p=0.002, interquartile range=0.0000**
**Roberta**	**z=2.567, p=0.010, interquartile range=0.375**	**z=3.162, p=0.002, interquartile range=0.000**	**z=2.227, p=0.023, interquartile range=0.242**	**z=3.051, p=0.002, interquartile range=0.000**
**Robinia**	**z=2.828, p=0.005, interquartile range=0.167**	**z=3.162, p=0.002, interquartile range=0.000**	**z=2.919, p=0.003, interquartile range=0.167**	**z=3.162, p=0.002, interquartile range=0.000**
**Robiola**	**z=2.972, p=0.003, interquartile range=0.083**	**z=3.051, p=0.002, interquartile range=0.000**	**z=3.051, p=0.002, interquartile range=0.000**	**z=3.162, p=0.002, interquartile range=0.000**
**Robot**	**z=3.051, p=0.002, interquartile range=0.000**	**z=3.162, p=0.002, interquartile range=0.000**	**z=3.051, p=0.002, interquartile range=0.000**	**z=3.162, p=0.002, interquartile range=0.000**

**Rucola**	**z=2.862, p=0.004, interquartile range=0.208**	**z=3.162, p=0.002, interquartile range=0.000**	**z=3.162, p=0.002, interquartile range=0.000**	**z=3.051, p=0.002, interquartile range=0.000**
**Sandokan**	**z=2.877, p=0.004, interquartile range=0.167**	**z=3.162, p=0.002, interquartile range=0.000**	**z=3.051, p=0.002, interquartile range=0.000**	**z=2.972, p=0.003, interquartile range=0.042**
**Saroma**	**z=2.773, p=0.006, interquartile range=0.375**	**z=3.051, p=0.002, interquartile range=0.000**	**z=2.850, p=0.004, interquartile range=0.333**	**z=3.162, p=0.002, interquartile range=0.000**
**Totò**	**z=3.162, p=0.002, interquartile range=0.000**	**z=3.162, p=0.002, interquartile range=0.000**	**z=3.162, p=0.002, interquartile range=0.000**	**z=2.877, p=0.004, interquartile range=0.167**
**Vispo**	**z=2.877, p=0.004, interquartile range=0.167**	**z=3.162, p=0.002, interquartile range=0.000**	**z=1.968, p=0.049, interquartile range=0.375**	**z=3.162, p=0.002, interquartile range=0.000**

Table S5. Control trials for quantitative discrimination with highly preferred food (HH *vs*. H). The table reports the individual results of Wilcoxon's single-sample test, including the p value, median, and interquartile range. Significant results are highlighted in bold

	**"HL^{+} 10"**	**"HL^{-} 10"**	**"HL^{+} 30"**	**"HL^{-} 30"**
**Cognac**	**z=2.972, p=0.003, interquartile range=0.083**	**z=3.051, p=0.002, interquartile range=0.000**	**z=3.162, p=0.002, interquartile range=0.000**	**z=3.162, p=0.002, interquartile range=0.000**
**Gal**	**z=2.889, p=0.004, interquartile range=0.333**	**z=3.162, p=0.002, interquartile range=0.000**	**z=2.972, p=0.003, interquartile range=0.083**	**z=3.162, p=0.002, interquartile range=0.000**
**Paprika**	**z=3.051, p=0.002, interquartile range=0.000**	**z=3.051, p=0.002, interquartile range=0.000**	**z=3.051, p=0.002, interquartile range=0.000**	**z=3.162, p=0.002, interquartile range=0.000**
**Roberta**	**z=3.051, p=0.002, interquartile range=0.000**	**z=3.051, p=0.002, interquartile range=0.000**	**z=2.755, p=0.006, interquartile range=0.333**	**z=3.051, p=0.002, interquartile range=0.000**
**Robinia**	**z=3.051, p=0.002, interquartile range=0.000**	**z=3.162, p=0.002, interquartile range=0.000**	**z=3.051, p=0.002, interquartile range=0.000**	**z=3.051, p=0.003, interquartile range=0.000**
**Robiola**	**z=3.051, p=0.002, interquartile range=0.000**	**z=2.972, p=0.003, interquartile range=0.083**	**z=3.162, p=0.002, interquartile range=0.000**	**z=3.051, p=0.002, interquartile range=0.000**
**Robot**	**z=2.972, p=0.003, interquartile range=0.083**	**z=3.162, p=0.002, interquartile range=0.000**	**z=3.162, p=0.002, interquartile range=0.000**	**z=3.162, p=0.002, interquartile range=0.000**

**Rucola**	**z=2.919, p=0.003, interquartile range=0.333**	**z=3.162, p=0.002, interquartile range=0.000**	**z=3.162, p=0.002, interquartile range=0.000**	**z=3.162, p=0.002, interquartile range=0.000**
**Sandokan**	**z=3.051, p=0.002, interquartile range=0.000**	**z=3.162, p=0.002, interquartile range=0.000**	**z=3.162, p=0.002, interquartile range=0.000**	**z=3.162, p=0.002, interquartile range=0.000**
**Saroma**	**z=2.889, p=0.004, interquartile range=0.333**	**z=3.051, p=0.002, interquartile range=0.000**	**z=2.972, p=0.003, interquartile range=0.083**	**z=2.972, p=0.003, interquartile range=0.083**
**Totò**	**z=3.162, p=0.002, interquartile range=0.000**	**z=3.162, p=0.002, interquartile range=0.000**	**z=2.972, p=0.003, interquartile range=0.083**	**z=3.051, p=0.002, interquartile range=0.000**
**Vispo**	**z=3.051, p=0.002, interquartile range=0.000**	**z=3.051, p=0.002, interquartile range=0.000**	**z=2.972, p=0.003, interquartile range=0.083**	**z=3.162, p=0.002, interquartile range=0.000**

Table S6. Control trials for quantitative discrimination with intermediate-preferred food (LL+ *vs*. L+) and low-preferred food (LL- *vs*. L-). The table reports the individual results of Wilcoxon's single-sample test, including the p value, median, and interquartile range. Significant results are highlighted in bold

	**“HL^+ 10”**	**“HL^- 10”**	**“HL^+ 30”**	**“HL^- 30”**
**Cognac**	**z=2.889, p=0.004, interquartile range=0.333**	**z=2.919, p=0.003, interquartile range=0.333**	**z=3.051, p=0.002, interquartile range=0.000**	**z=2.807, p=0.005, interquartile range=0.333**
**Gal**	**z=3.162, p=0.002, interquartile range=0.000**	**z=2.972, p=0.003, interquartile range=0.083**	**z=3.162, p=0.002, interquartile range=0.000**	**z=2.919, p=0.003, interquartile range=0.333**
**Paprika**	**z=2.919, p=0.003, interquartile range=0.333**	**z=3.162, p=0.002, interquartile range=0.000**	**z=3.051, p=0.002, interquartile range=0.000**	**z=3.051, p=0.002, interquartile range=0.000**
**Roberta**	**z=3.162, p=0.002, interquartile range=0.000**	**z=3.051, p=0.002, interquartile range=0.000**	**z=3.162, p=0.002, interquartile range=0.000**	**z=3.051, p=0.002, interquartile range=0.000**
**Robinia**	**z=3.162, p=0.002, interquartile range=0.000**	**z=2.919, p=0.003, interquartile range=0.333**	**z=3.162, p=0.002, interquartile range=0.000**	**z=3.051, p=0.002, interquartile range=0.000**
**Robiola**	**z=3.162, p=0.002, interquartile range=0.000**	**z=2.772, p=0.006, interquartile range=0.333**	**z=3.162, p=0.002, interquartile range=0.000**	**z=2.249, p=0.024, interquartile range=0.667**
**Robot**	**z=3.162, p=0.002, interquartile range=0.000**	**z=2.972, p=0.003, interquartile range=0.083**	**z=3.162, p=0.002, interquartile range=0.000**	**z=2.920, p=0.003, interquartile range=0.083**

**Rucola**	**z=2.972, p=0.003, interquartile range=0.083**	**z=3.162, p=0.002, interquartile range=0.000**	**z=2.972, p=0.003, interquartile range=0.083**	**z=3.162, p=0.002, interquartile range=0.000**
**Sandokan**	**z=3.051, p=0.002, interquartile range=0.000**	**z=3.162, p=0.002, interquartile range=0.000**	**z=3.162, p=0.002, interquartile range=0.000**	**z=3.162, p=0.002, interquartile range=0.000**
**Saroma**	**z=2.560, p=0.010, interquartile range=0.417**	**z=2.972, p=0.003, interquartile range=0.083**	**z=2.807, p=0.005, interquartile range=0.333**	**z=2.532, p=0.011, interquartile range=0.417**
**Totò**	**z=3.051, p=0.002, interquartile range=0.000**	**z=3.162, p=0.002, interquartile range=0.000**	**z=2.972, p=0.003, interquartile range=0.083**	**z=3.051, p=0.002, interquartile range=0.000**
**Vispo**	**z=3.162, p=0.002, interquartile range=0.000**	**z=3.162, p=0.002, interquartile range=0.000**	**z=3.162, p=0.002, interquartile range=0.000**	**z=2.972, p=0.003, interquartile range=0.083**

Table S7. Control trials for food preference maintenance (H *vs*. L+ and H *vs*. L-). The table reports the individual results of Wilcoxon's single-sample test, including the p value, median, and interquartile range. Significant results are highlighted in bold

	"HL^{+} 10"	"HL^{-} 10"	"HL^{+} 30"	"HL^{-} 30"
**Cognac**	**z=3.051, p=0.002, interquartile range=0.000**	**z=2.913, p=0.004, interquartile range=0.286**	**z=2.972, p=0.003, interquartile range=0.042**	**z=2.972, p=0.003, interquartile range=0.042**
**Gal**	**z=-2.783, p=0.005, interquartile range=0.333**	**z=2.970, p=0.003, interquartile range=0.036**	z=-1.839, p=0.066, interquartile range=0.042	**z=3.051, p=0.002, interquartile range=0.000**
**Paprika**	**z=2.859, p=0.004, interquartile range=0.167**	**z=2.825, p=0.005, interquartile range=0.286**	z=-1.025, p=0.305, interquartile range=0.542	**z=2.972, p=0.003, interquartile range=0.042**
**Roberta**	**z=-2.783, p=0.005, interquartile range=0.333**	**z=3.162, p=0.002, interquartile range=0.000**	**z=-2.920, p=0.003, interquartile range=0.042**	**z=3.162, p=0.002, interquartile range=0.000**
**Robinia**	**z=2.369, p=0.018, interquartile range=0.375**	**z=3.162, p=0.002, interquartile range=0.000**	z=1.951, p=0.051, interquartile range=0.250	**z=3.051, p=0.002, interquartile range=0.000**
**Robiola**	z=-1.137, p=0.256, interquartile range=0.250	**z=3.051, p=0.002, interquartile range=0.000**	z=0.413, p=0.680, interquartile range=0.500	**z=3.162, p=0.002, interquartile range=0.000**
**Robot**	**z=2.767, p=0.006, interquartile range=0.208**	**z=3.162, p=0.002, interquartile range=0.000**	**z=2.689, p=0.007, interquartile range=0. 375**	**z=3.162, p=0.002, interquartile range=0.000**

**Rucola**	**z=-2.711, p=0.007, interquartile range=0.208**	**z=2.970, p=0.003, interquartile range=0.036**	z=1.800, p=0.072, interquartile range=0.250	**z=2.587, p=0.010, interquartile range=0.375**
**Sandokan**	z=0.154, p=0.878, interquartile range=0.387	**z=2.869, p=0.004, interquartile range=0.196**	**z=2.136, p=0.033, interquartile range=0.333**	**z=2.823, p=0.005, interquartile range=0.208**
**Saroma**	z=1.179, p=0.238, interquartile range=0.500	**z=3.051, p=0.002, interquartile range=0.000**	z=-0.103, p=0.918, interquartile range=0.375	**z=3.162, p=0.002, interquartile range=0.000**
**Totò**	**z=2.877, p=0.004, interquartile range=0.167**	**z=2.765, p=0.006, interquartile range=0.196**	z=1.951, p=0.051, interquartile range=0.250	z=0.820, p=0.412, interquartile range=0.542
**Vispo**	**z=-2.831, p=0.005, interquartile range=0.333**	**z=3.162, p=0.002, interquartile range=0.000**	**z=-2.823, p=0.005, interquartile range=0.208**	**z=3.162, p=0.002, interquartile range=0.000**
